# Health problems and healthcare service utilisation amongst homeless adults in Africa- a scoping review

**DOI:** 10.1186/s12889-020-08648-y

**Published:** 2020-05-01

**Authors:** Benedict Osei Asibey, Elizabeth Conroy, Brahmaputra Marjadi

**Affiliations:** 1 Translational Health Research Institute, Western Sydney University, Locked Bag 1797, Penrith, New South Wales 2751 Australia; 2 School of Medicine, Western Sydney University, Locked Bag 1797, Penrith, New South Wales 2751 Australia

**Keywords:** Homelessness, Needs, Health problems, Health care, Utilisation, Africa

## Abstract

**Background:**

Homelessness is a growing concern as it affects a large number of people worldwide. Individuals and families experiencing homelessness are vulnerable in terms of health and underutilise health services. Despite being a global problem, not much is known about the range and breath of literature exploring health problems and health care service utilisation among homeless adults in Africa.

**Objectives:**

To identify the nature and scope of existing evidence on physical and mental health, and health service utilisation among homeless adults in Africa. The review aimed to examine how research is conducted, identify gaps, guide future research, and make recommendations for development and implementation of policies and practices.

**Methods:**

A search of articles and reports involving six databases: Scopus, MEDLINE, CINAHL, PubMed, African Journal Online, and Google Scholar was conducted from June 2018 to February 2019. Studies published between 1980 and 2019 that examined the health problems and health service utilisation among homeless adults in Africa were considered. Manual search in reference lists and grey literature was also done to add reports. Data was extracted manually using a charting developed. A descriptive analysis and narrative synthesis were performed.

**Results:**

Of 761 records found, 14 satisfied the pre-determined inclusion and exclusion criteria. Three themes emerged from the studies: Physical health problems, mental health problems; and healthcare services utilisation. Of the 14 included studies, nine studied and reported physical health problems such as sexually transmitted infections, injuries and disabilities, respiratory and cardiac diseases. Five studies explored mental health problems such as psychotic disorders, mood disorders, self-harm and suicidal behaviour. Only five studies investigated utilisation of different types of health care services among homeless people.

**Conclusions:**

Evidence shows that homeless adults suffered from a range of physical and mental health problems, and underutilisation of health care services. However, there is lack of information on the complex interrelationship between homelessness and health, as well as differences in prevalence of health problems among the various sub-groups of homeless. There is also lack of information regarding utilisation other important healthcare services such as mental health services, alcohol and drugs services, and accident and emergency service, and future researches should address that. Also, attention should be given to intervention models for complex and effective physical and psychiatric care as well as social support to address the homeless people’s health vulnerabilities.

## Background

Homelessness affects a growing number of people worldwide, and presents a complex challenge for policymakers and service providers. Various United Nations estimates show that between 100 to 150 million people experience different forms of homelessness worldwide [1–3]. This is a concern because the homeless population is susceptible to various health problems due to their sleeping conditions [4–10], and  they are a marginalised group with poor access and low usage of health care services [5].

African countries generally have a housing challenge manifested in high housing deficits, higher prices, growing slums, overcrowding, and homelessness particularly in urban areas [11]. Although official data on homelessness in Africa are scarce and definitions and measurements vary from country to country, available country estimates show a high prevalence. For instance, in Zimbabwe 1.2 to 1.5 million people are estimated to be homeless, defined as living in informal residential settlements [12]. An estimated 24.4 million people are homeless in Nigeria defined to include both rough sleepers and internally displaced people [13]. Egypt has 12 million homeless people including rough sleepers, individuals living in unsuitable housing such as shacks and kiosks, and in public institutions [14, 15]. South Africa has estimated 200,000 homeless people, both sheltered and unsheltered [16], and there are 100,000 roofless people in Ghana [17]. The high prevalence of homelessness in Africa is caused by a number of factors, and migration is key [18, 19]. Driven by poverty and other socio-economic difficulties beyond the individual’s control, people migrate to cities mostly unprepared and unaware of the risks involved. The result is a rapid growth of the low income urban population, and coupled with housing deficits and high prices result in rough sleeping and overcrowded squats and slums [18, 19].

In some parts of Africa, civil conflicts, political unrest, and activities of terrorist groups are destroying lives and homes, displacing people, and rendering millions of people homeless [20]. The actions of Boko Haram in Nigeria has led to an estimated 650,000 people being internally displaced [20]. Past and current civil conflicts, political unrest and terrorism in Somalia, Congo, Sudan, Rwanda, Ethiopia, Burundi, and Uganda have caused many displaced and homeless people, both internally and in other neighbouring countries. Approximately 55% of refugees in Kenya, for example, originate from Somalia, 25% originate from South Sudan, 9% originate from Congo, and 6% originate from Ethiopia [20]. Many of the refugees find their way into slums, squats and the streets.

Homelessness is associated with a lot of problems and the consequences of homelessness for the affected individuals and communities are damaging. Homeless people experience poor health, suffer from a range of physical and mental problems related to their sleeping conditions and the environment in which they live. Substance abuse, violence, and malnourishment are overrepresented in the homeless population [7, 11]. Within Africa, the prevalence of physical and mental health problems in the homeless population may differ from country to country depending on treatment coverage and support services available, as well as differences in family support systems and cultural responses to illnesses [21]. In terms of health care access and usage, even basic services can be beyond their means [22]. Barriers such as low incomes, lack of health insurance, poor accessibility, and stigma and discrimination mean homeless people have poor engagement with healthcare services [22–24]. Their delayed clinical presentation leads to increased reliance on emergency departments and higher rates of hospitalisation for preventable conditions [25]. From the society and community perspective, high prevalence of homelessness puts excessive pressure on scarce resources including health care services [11].

Health systems in most African countries are generally weak, and many have not attained equitable and sustainable access to well-functioning health systems [26]. Health services are mostly out-of-pocket and people access services only if they can pay. Few countries operate voluntary national, community-based, or private health insurance schemes [27–29], but most of these insurance schemes require paying a premium and annual renewals before patients can obtain subsidised care which makes the poor and vulnerable people including the homeless forgo using services [30]. Insurance schemes also mostly do not cover all services^,^ and registered patients are sometimes required to pay [31, 32]. Governments and the private sector make efforts to provide services including primary care, emergency services, maternal care, and mental health services. In spite of the progress in improving health outcomes, most African countries still face challenges in providing adequate healthcare services, particularly to the vulnerable. The number, quality, and competency of health care workers as a ratio to the population is low and countries still face higher burden of morbidity and mortality [26].

### Objective of the current review

Although health issues are an important focus for homelessness research and a substantial body of literature exploring health and health service usage among homeless people exists, little is known about the situation in Africa. In order to identify the nature and scope of existing evidence, identify gaps, guide future research, and recommend policies and interventions for implementation, a scoping review which is suitable for gathering information from many different sources was conducted. The review was guided by the overarching research question: What is the scope and nature of literature exploring the health problems and health care utilisation among homeless people in Africa? The specific objective of this review was to explore and summarise existing research on the physical and mental health illnesses experienced by homeless adults, and their health care service utilisation in Africa.

## Methods

A scoping review was conducted from June 2018 to February 2019 according to the guidelines in the Joanna Briggs Institute’s Preferred Reporting Items for Systematic reviews and Meta-Analyses extension for Scoping Reviews (PRISMA-ScR) checklist [33].

### Description of the study population

The population of interest in the review was the homeless adults 18 years and above. To be considered homeless, a person must meet one of the following criteria: (1) be rough sleeping, roofless, or in places not designed for shelter; (2) unstably residing in slums, shacks, and squats; (3) residing in a homeless shelter, crisis and emergency accommodations temporarily, and (5) be admitted into drug rehabilitation, psychiatric facilities or religious healing centres with immediate preceding homeless episode and/or no place of residence when discharged [34]. The definition of homelessness in this review excluded: (1) individuals living in accommodation with insecure tenancy, and (2) individuals living in slums/shanty towns but in stable accommodation.

### Information sources and search strategy

A search for articles was conducted on six electronic databases: Scopus, MEDLINE, CINAHL (EBSCO), PubMed, African Journal Online (AJOL), and Google Scholar. The search involved three steps: (a) initial search in MEDLINE to confirm search terms; (b) use of keywords and MeSH terms to search for articles across all databases (abstract and title search only); and (c) manual search of reference lists of included articles. A complete search strategy for Medline is provided in supplementary Table 1. Database search was accompanied by a search of grey literature using the internal search tools of websites of homeless charities, government and non-governmental institutions in Africa such as Department of Social Welfare in African countries. Websites of popular homeless NGOs such as Haven Night Shelter, Chance for Children, Remar Ghana and Cote D’Ivoire were also searched for grey literature.

The search terms and keywords are shown in Table 1. The thesaurus or controlled vocabulary of each database were searched. In addition to the database’s controlled vocabulary (where applicable), search keywords were used.

**Table 1:** Search Terms under Specific Areas

POPULATION	CONCEPT #1	CONCEPT #2	CONTEXT
**Homeless and street people**	**Health Problems**	**Health service utilisation**	**Africa**
Homeless Persons [Keyword]	Health [MeSH]	Utilisation [Keyword]	Africa [MeSH]
Homelessness [Keyword]	Health needs [Keyword]	Health Services [MeSH]	
Homeless youth [Keyword]	Health conditions [Keyword]	Health care [Keyword]	
Homeless family [MeSH]	Health status [MeSH]	Health delivery [MeSH]	
Homeless men [MeSH]	Physical health [Keyword]	Health facilities [MeSH]	
Homeless women [MeSH]	Mental health [MeSH]	Primary health care [MeSH]	
Homeless people [Keyword]	Poor health [Keyword)	Health service use [Keyword]	
Street People [Keyword]	Diseases [MeSH]	Health service Uptake [Keyword]	
Street dwellers [Keyword]	Infirmities [Keyword]	Health service seeking [Keyword]	
Sleeping rough [Keyword]	Comorbidities [MeSH]	Health service consumption [Keyword]	
Roofless [Keyword]	Morbidities [MeSH]	Health service usage [Keyword]	
Houseless [Keyword]	Illnesses [Keyword]		
	Ill-health [Keyword]		
	Sicknesses [Keyword]		
	Health problems [Keyword]		

### Study selection and eligibility criteria

All citations were uploaded into the Covidence online systematic review programme, which was used for the study selection. The selection of studies was undertaken in three phases of screening: (a) removing duplicates, (b) screening titles and abstract, and (c) screening full text articles. At each phase, the articles were compared against a pre-determined set of inclusion and exclusion criteria. To be included, a study:

- Must be conducted in Africa
- Must focus on homeless adults (described under the section on study population).
- Must focus on physical or mental health
- Must be published in the English language from 1980 to 2019

All excluded papers were pointed out along with reasons, as follows:

- Not conducted in Africa
- Wrong population (not homeless adults)
- Not focused on health, and
- Not published in the English Language from 1980 to 2019

### Data items and charting

A data charting form was developed and used to manually capture data relevant to the review objective. The form had the following sections: (a) author(s)/publication year, (b) geographical location, (c) study design, (d) study population, (e) sample size and (f) summary of key findings. Data charting was done by authors in duplicate. The following were charted from the included studies: documented chronic and acute physical health problems; documented chronic and acute mental health problems; health seeking behaviour including frequencies and types of healthcare service utilised.

### Data synthesis and reporting

A thematic analysis of the results from the studies was conducted. The process of data analysis identified three main themes concerning health problems and health service usage: physical health, mental health, and healthcare service usage. Studies have been grouped according to these three themes. Finally, a narrative synthesis and interpretation of the data was undertaken.

## Results

### Selection of sources of evidence

**Fig. 1:**
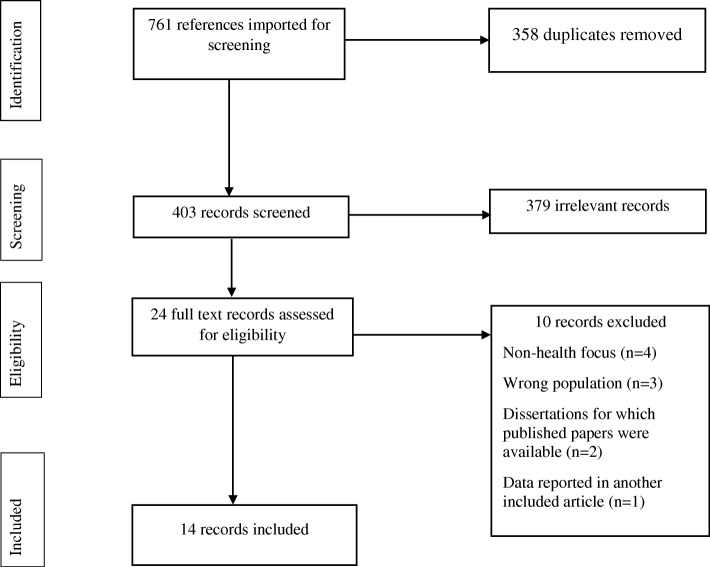
PRISMA Flow Chart

Out of the 761 records identified, the full texts of 24 articles were screened for inclusion, and 14 records satisfied the inclusion criteria (Fig. 1).

### Characteristics of the included studies

Table 2 summarises the characteristics of the included studies. The records were mostly published in the last decade (2009–2019). Apart from one study from North Africa [40], all other studies were conducted in sub-Sahara Africa predominantly South Africa [30, 36–38, 44] and Ethiopia [11, 39, 42, 45, 46]. The remaining studies were conducted in Nigeria [35], Ghana [41], and Mozambique [43].

**Table 2:** Characteristics of the Included Studies

Reference	Country	Aim/Purpose	Sample type	Sample Size	Sampling Method	Study Design	Data Collection Methods
[11] Ayano et al. (2017)	Ethiopia	Examine the prevalence of mental, neurologic, and substance use (MNS) disorders among street homeless people	Roofless people with overt and observable psychopathology	456	Multistage cluster Sampling	Quantitative (community-based cross-sectional study)	Survey questionnaire
[30] Wentzel & Voce (2012)	South Africa	Describe health seeking behaviours and experiences of homeless people	Homeless persons in shelters	18	Purposive	Qualitative	Semi-structured interview
[35] Abdu et al. (2013)	Nigeria	Examine prevalence of common diseases among homeless people	Homeless street people	65	Not reported	Quantitative	Clinical examinations and questionnaire
[36] Moyo et al. (2015)	South Africa	Examine the social and health situation of homeless people with mental illness	Homeless persons with suspected mental illness	18	Purposive	Qualitative (exploratory design)	In-depth interviews
[37] Olufemi (1999)	South Africa	Explore the types of diseases prevalent among the street homeless women and their use of healthcare services.	Homeless street women	88	Purposive	Qualitative	In-depth Interview
[38] Seager & Tamasane (2010)	South Africa	Explore the health status and health service needs of homeless people	Homeless street people and people in shelters	1247	Purposive and convenient sampling	Mixed	Structured interviews, FGDs
[39] Semunigus et al. (2016)	Ethiopia	Determine the prevalence and associated factors of smear positive (PTB) among homeless individuals.	Homeless individuals	361	Active screening to identify PTB suspects	Quantitative (community based cross-sectional study)	Clinical examination and survey questionnaire
[40] Khelil et al. (2017)	Tunisia	Analyse causes of death occurring among homeless persons.	Cases of homeless mortality	152	Census	Quantitative (descriptive, retrospective)	Clinical database search
[41] De-Graft Aikins & Ofori-Atta (2007)	Ghana	Explore everyday experiences and mental health of individuals living in squatter settlements	Homeless adults living in squats	28	Purposive	Qualitative	In-depth interview
[42] Fekadu et al. (2014)	Ethiopia	Determine the burden of mental health problems and associated unmet needs	Street homeless people 18 years and above	217	Double stage sampling	Quantitative (Cross-sectional Study)	Clinical examination and Survey questionnaire
[43] Gouveia et al.(2014)	Mozambique	Describe the mental health status of the homeless people.	Homeless people with apparent mental illness	71	Not reported	Quantitative (descriptive)	Structured clinical interview, survey questionnaire
[44] Lohrmann et al. (2012)	South Africa	Investigate the HIV prevalence and risk factors among urban homeless individuals.	Adults from a homeless clinic	136	Census of soup kitchen attendants	Quantitative (Cross-sectional Study)	Clinical HIV test, demographic survey
[45] Megabiaw (2012)	Ethiopia	Assess awareness and usage of modern contraceptives among street women	Street women	204	Cluster	Quantitative (Cross-sectional Study)	Survey Questionnaire
[46] Moges et al. (2006)	Ethiopia	Assess the prevalence of HIV and intestinal parasites among street dwellers	Street dwellers	404	Simple random	Quantitative (Cross-sectional Study)	Clinical examination and questionnaire

The studies mostly used primary data. One study was conducted through database analysis [40]. Studies were mostly quantitative utilising clinical examinations, surveys, and registry data. The four qualitative studies obtained data using interviews and focus group discussions [30, 36, 37, ">41]. The sample size for quantitative studies ranged from 65 to 404, the qualitative samples ranged from 18 to 88, and the only mixed methods study had a total sample size of 1287. Studies mostly drew sample from rough sleepers [11, 35–37, 39, 42, 43, 46]. Studies also drew samples from people in squats and slums [41], and people in accommodation services including homeless clinics, shelters and drop-in centres [30, 43, 44]. One study sampled from both streets and shelters [38], and one study failed to mention category of homeless people studied [40]. Only two study assessed the duration of homelessness and found that most participants were chronically homeless [11, 39]. Two studies sampled only women [37, 45] and the remainder recruited both males and females.

### Narrative synthesis

Three different themes emerged in the analysis of included studies: (1) Physical health problems; (2) Mental health problems; and (3) Underutilisation of health care services. All records associated with various themes, and related sub-themes are summarised in Table 3.

**Table 3:** Themes and Sub-Themes Represented by Included Studies

Theme	Key Findings	Included Studies
Physical Health Problems (*n* = 9)	Physical Injuries (*n* = 2)	[30, 38]
	HIV (*n* = 6)	[30, 36, 38, 39, 44, 46]
	Visual Impairments (*n* = 2)	[35, 37]
	Hypertension (*n* = 1)	[35]
	Skin diseases (*n* = 2)	[35, 38]
	Physical disabilities (*n* = 2)	[35, 38]
	Coughing (*n* = 3)	[35, 37, 46]
	Stomach diseases (*n* = 2)	[37, 46]
	Tuberculosis (*n =* 3)	[37–39]
	Dental Problems (*n* = 1)	[37]
	Arthritis (*n* = 1)	[37]
	Diabetes (*n* = 1)	[37]
	Pneumonia (*n* = 1)	[37]
	Asthma (*n* = 1)	[37]
	Severe headache (*n* = 2)	[37, 46]
	Cardiac diseases (*n* = 1)	[40]
	Pulmonary diseases (*n* = 1)	[40]
	Fever (*n* = 1)	[46]
	Malaise (*n* = 1)	[46]
Mental Health Problems (*n* = 5)	Mental neurologic and substance use (MNS) disorder (*n* = 1)	[11]
	Psychotic Disorders (*n* = 3)	[11, 42, 43]
	Depression (*n* = 1)	[38]
	Schizophrenia (*n* = 3)	[38, 42, 43]
	Bipolar and mood disorders (*n* = 2)	[38, 42]
	Psychological distress (*n* = 2)	[41, 42]
	Anxiety (*n* = 1)	[41]
	Alcohol use disorders (*n* = 1)	[42]
	Suicide thoughts and attempts (*n* = 1)	[42]
	Intellectual disability (*n* = 1)	[43]
	Mental and behavioural disturbance (*n* = 1)	[43]
Healthcare Service Utilisation (*n* = 5)	Low hospital and clinic care utilisation (*n* = 4)	[30, 35, 37, 38]
	Complementary care usage (Drug vendors, traditional healers) (*n* = 2)	[35, 38]
	Self-medication (*n* = 1)	[35]
	Lifetime and current usage of modern contraceptives (*n* = 1)	[45]

### Theme 1: physical health of homeless adults

Overwhelmingly, studies found various physical health problems among homeless adults in African countries. The nine studies that measured physical health reported a range of illnesses. One study specifically looked at smear positive pulmonary tuberculosis [39], and two studies specifically investigated HIV [44, 46]. The other six studies openly explored physical health and identified three groups of health problems: respiratory illnesses, cardiovascular diseases, and disabilities and injuries [30, 35–38, 40].

Respiratory illnesses were reported in six studies conducted in South Africa [37, 38], Ethiopia [39, 46], Nigeria [35], and Tunisia [40]. Respiratory and related illnesses reported include pneumonia, asthma and bronchitis [37], pulmonary diseases [40], tuberculosis [37–39], and severe coughing [35, 37, 46]. The estimated prevalence of respiratory illnesses among the participants ranged from 2.6 to 39.2% (see supplementary Table 2). These six studies were conducted in different settings, used different samples and diagnostic approaches. Two studies primarily focused on respiratory problems such as smear positive pulmonary tuberculosis [39], and tuberculosis and coughing in related to HIV [46] based on street samples, and assessed diseases using clinical examinations. The other four studies generally measured health status and documented all respiratory and related problems found [35, 37, 38, 40]. Three of the four general health studies were based on street samples [35, 37, 38], and one on shelter dwellers [38]. Diagnostic approaches used include clinical tests [35, 40], questionnaires, and in-depth interviews [37, 38].

Prevalence of cardiovascular diseases was presented in two studies in Nigeria [35] and Tunisia [40]. Using primary data obtained from clinical examination, the Nigerian study found 35% of the sampled 65 street dwellers had hypertension. The Tunisian study used secondary data consisting of results from clinical tests for causes of 152 homeless mortalities and found 26% of 152 deaths were caused by cardiac diseases.

Physical disabilities and injuries were presented in four studies based on street sample [30, 35, 37, 39]. Using FGDs and questionnaires studies identified and assessed prevalence of injuries and disabilities as key health problems. Prevalence of injuries and disabilities reported in studies ranged from 19% to (37%). Physical disabilities reported in studies include lost limbs, paraplegia, and inability to walk, visual impairments [35, 37, 38]. Studies also reported different kind of injuries such as broken legs and arms, cuts, ankle sprain, and knee injury [30, 38].

In addition to the physical health problems mentioned above, HIV was presented in six studies including four in South Africa [30, 36, 38, 44] and two in Ethiopia [39, 46]. In the six studies, four generally examined the health of homeless adults and reported HIV prevalence [30, 36, 38, 39]. For example, using clinical test one Ethiopian study found 6.3% of the 361 street dwellers were HIV positive [39]. Apart from the four general health studies that found HIV, two studies specifically focused on HIV among homeless people in South Africa [44] and Ethiopia [46]. The South African study was based on sample of 136 people in homeless clinics, whilst the Ethiopian study sampled 404 street dwellers. Both studies, regardless of settings and subgroups of homeless people sample, used various clinical tests to diagnose HIV, with the estimated prevalence varying from 7 to 24% [44, 46].

Apart from the above-mentioned categories of health problems reported in included studies, other health problems such as stomach diseases among street and shelter residents were reorted [37, 38]. Skin diseases such as scabies, chicken pox, and rashes [38], headache [37, 46], malaise [46], diabetes, arthritis, and dental problems [37] were also reported among both street dwellers and people in shelters.

### Theme two: mental health of homeless adults

Five studies explored and reported a range of mental health problems across four sub-Saharan African countries [11, 38, 41–43]. The studies identified three groups of mental health problems: psychotic disorders, mood disorders, self-harm and suicidal behaviour, and intellectual disability. One of the five studies looked at general health and documented any illness reported including mental disorder [38]. The other four studies specifically examined mental disorders [11, 41–43], including one that specifically looked at mental, neurologic, and substance use disorder [11].

Four studies measured psychosis [11, 38, 42, 43]; three of which were based only on street samples [11, 42, 43] and one sampled from both streets and shelters [38]; with sample sizes ranging from 71 to 1247. Regardless of differences in setting and sample sizes, three studies measured psychosis using standardised clinical assessments such as the WHO Self Reporting Questionnaire (SRQ20) [11], Psychosis Screening Questionnaire (PSQ) [42], and Mini International Neuropsychiatric Interview (MINI) [43], and prevalence estimates ranged from 68 to 88% (see supplementary Table 2). One study which was a general health study determined prevalence of psychosis using survey interviews and reported a prevalence of 6% [38]. Although settings and sample sizes differed, studies based on street samples alone [11, 42, 43] reported higher prevalence of psychosis (see supplementary Table 3).

Depression and psychological stress were reported in three studies [38, 41, 42]. The estimated prevalence among the participants ranged from 58 to 74% (see supplementary Table 3). These three studies were conducted in different settings and used different samples and diagnostic approaches. Only one of these studies measured depression and psychological distress using a standardised clinical assessment (K10) [42]. The two other studies used surveys [38] and interviews [41]. One study first identified depression and psychological distress as a mental health problem using FGDs involving 24 homeless people in South African study. Then, using surveys involving 1247 participants, the study determined the prevalence of depression and distress among the homeless people using self-reports [38]. Another study in Ghana used episodic interviews for 28 squatters in Accra, and found psychological stresses commonly reported [41].

Bipolar and mood disorders were assessed in two studies, one among street dwellers and shelter residents [38] and one based on only street dwellers [42]. One study used self-reported survey questionnaires to document participants experience in the past 3 months [38], and the other study used a clinical assessment (specific tool not reported) [42]. Both studies found low prevalence of bipolar disorders among the homeless adults.

Only one study reported on self-harm and suicidal behaviour among a sample of street dwellers [42]. The study used a survey that asked whether the participant had experienced the wish to die or had suicidal thoughts, and whether the participant had attempted suicide in the past 1 month. The study reported 42% of the sampled street dwellers had a persistent wish to die and 15% had attempted suicide in the past 1 month. Lastly, one study specifically measured mental, neurologic and substance use (MNS) disorders, among roofless people who had overt and observable psychopathology [11]. Participants were initially screened using the SRQ20, ASSIST and PSQ and mental, neurologic and substance use disorders were then confirmed for 456 participants using a structured clinical interview based on the DSM-IV criteria. The study reported a 92% prevalence of mental, neurologic, and substance use disorders (see supplementary Table 3).

### Theme 3: health service utilisation

The homeless adults’ health service utilisation was explored in five studies [30, 35, 37, 38, 45]. Only one study specifically assessed utilisation of modern contraceptives among street women [45]. The other four studies generally looked at hospital and clinic services and complementary services. Studies reported underutilisation of the following health services: 1) hospital and clinic services, 2) complementary services, and 3) modern contraceptives.

#### Hospital and clinic services

Four studies explored homeless people’s utilisation of hospital and clinic services [30, 35, 37, 38]. Two of the studies used in-depth interviews and found that homeless people underutilise hospitals and clinic services [30, 37]. The two other studies used survey questionnaires and reported low prevalence of utilisation of hospital and clinic services (ranging from 9.3% 4 25%), although assessment period ranged from 3 months [38] to lifetime [35].

#### Complementary services

Only two studies elaborated on the use of complementary service among both street dwellers and shelter people; one was quantitative [35] and the other was mixed-methods [38]. Both studies used questionnaires to assess utilisation of health services and found a low use of complementary health services among the homeless adults. One study in Nigeria [35] reported that 15% of participants had used traditional medicines or visited traditional healers and 5% had practised self-medication with herbal medicines in their lifetime [35]. The study in South Africa also reported that 7% of the participants had used herbal medicine or visited a traditional healer in the preceding 3 months [38] (see supplementary Table 4).

#### Modern contraceptives

One study specifically investigated the prevalence of utilisation of contraceptives among female adult street dwellers in Ethiopia [45]. The study assessed and reported self-reported lifetime and current utilisation of modern contraceptives such as injectable, pills, Norplant, and condoms. Use of modern contraceptives was estimated to be low. Approximately 47% of the homeless women reported a lifetime usage of modern contraceptives, and 34% reported current usage (see supplementary Table 4).

## Discussions

This review is the first to explore the literature relating to the critical issues of health and health service utilisation among homeless adults in Africa. The review identified studies with different settings, focus, and design, and there was a heterogeneity of study samples and methods employed to assess both physical and mental illnesses, and health service utilisation. Studies’ findings converge to indicate that homeless adults in African countries have high burdens of physical and mental ill-health and underutilise health care services.

A key theme in this review is that studies explored and reported a range of chronic and acute physical health problems among homeless people, including HIV, respiratory diseases, physical disabilities and injuries, cardiac diseases, and skin diseases. Studies in African countries commonly focused on HIV. This is possibly due to the high burden of the disease on the continent particularly in sub-Sahara African countries such as South Africa which has the highest prevalence of HIV in the world. Therefore, a greater number of HIV studies are funded and conducted both in the general population and among vulnerable populations including the homeless.

Mental health problems were commonly reported to be quite high regardless of the focus of the studies. This high prevalence may be due to three factors. First, the rough living condition including stress, trauma, and victimisation could directly cause mental health problems. Second, the poor support available for mental health may have resulted in people with mental illnesses becoming homeless [21]. Third, some studies may have selection bias toward recruiting participants with suspected or confirmed mental health problems. In spite of these limitations in some studies, there remains strong evidence for an association between homelessness and mental ill-health, because homeless experience such as constant fear, stress, danger and victimisation results in people becoming emotionally distressed, and unstable mental condition may result in a person becoming homeless [21].

A key point of divergence among the studies was the many different definitions of homelessness. Many of the studies sampled rough sleepers, roofless individuals, and individuals in squats and shacks; and institutional homeless people were inconsistently included. Another problematic point of divergence among the studies was their varying methods to assess physical and mental health problems, from clinical examinations and standardized instruments to self-reports. The use of clinical examinations and standardized instruments tended to result in higher prevalence than self-reports.

Although studies’ focus and measurement approaches differed, physical health and mental health were more commonly found among the rough sleepers compared to homeless shelter dwellers. Moreover, health problems among people in homeless shelters and crisis accommodations were likely underestimated because few studies sampled from shelters. This is so because unsheltered people including people residing in squats and shacks, and streets far outnumber shelter dwellers and people in emergency accommodations in Africa. Some African countries do not even have homeless shelters, and homelessness is basically defined to mean unsheltered people or people without roofs [18]. Also, people with observable mental illnesses happen to reside on the streets more than they are found in few shelters available in African countries. Sleeping rough and its associated vulnerabilities is psychologically stressful and can be associated with mental disorders such as anxiety, and suicidal thoughts [47].

The review found low health service utilisation, but similar to findings in other aspects in this review the findings from various studies were not directly comparable. In order to explore this matter further in future research, we recommend three improvements. First, future studies of service utilisation need to ensure a balance representation of street dwellers and shelter dwellers. Second, the time frame used in asking service utilisation needs to be standardised, for example in the past 6 months, bearing in mind the risk of recall bias. Third, the use of mixed-methods research may offer benefit from the combination of in-depth insight from qualitative methods and the generalisability of quantitative methods.

What emerges from the health service utilisation theme is the need to recognise and address the various barriers that limit homeless adults in their care seeking, including a number of socio-economic barriers [30]. Healthcare is expensive in Africa and the out-of-pocket system that exist in many counties [24, 25] requires that people can access healthcare services only if they can pay, which makes it access difficult for the low the income people [30] including the homeless. Even in countries where there are health insurance policies, not all health needs are subsidised [48], and poor people mostly cannot afford to pay premium and periodic renewal of insurance to receive subsidised care [26]. Fear of stigmatisation and discrimination by health care providers may also explain homeless people’s underutilisation of healthcare services in Africa. Homeless people are treated with less respect and described with stigmatised identities such as criminality, substance intake, and mental illness [47]. This may lead to negative feelings and experiences of being judged whenever they access health services [30, 37].

### Gaps in literature

One notable gap in the records identified is the lack of information regarding the complex interrelationship and direction of causality between homelessness and physical and mental health. Understanding the underlying interrelationships between homelessness and poor health might help in the design of preventive interventions and reduce co-occurrences. No study has also focused on the difference in prevalence of health problems among the sub-groups of homeless people, and what factors account for such differences. Better understanding of the differences in prevalence of health problems among the various sub-groups of homeless might help in the design of intervention models for complex health presentations and effective physical and psychiatric care as well as social support for this population.

There is also lack of information regarding utilisation of some specific healthcare services which are important to the homeless population. Homeless people’s use of mental health services, alcohol and drugs services, and accident and emergency service have not been studied. Utilisation of sexual and reproductive health services, maternal health services, and traditional/spiritual health services has also not been studied. A rigorous evidence base on how homelessness affects access and use of various health services in Africa is needed, particularly in sub-Sahara Africa, which carries the greatest homelessness problem, disease burden, and poverty.

### Limitations

The search for research articles in the electronic databases are not error free and the search of grey literature was not systematic. Included studies covered only six out of 54 African countries and no study performed a multi-country analyses. Caution is needed when making generalisations from the review findings to homeless adult populations across Africa. The scope and method of the included studies also differed. There were differences in the quantitative studies in terms of study population, sampling techniques, sample size, and measurement methods. Also, interpretation of the results of this review need to be considered with the knowledge that scoping reviews do not screen for quality of studies.

The review considered only records reported in English published since 1980. Broadening the scope of the review in terms of languages would have yielded additional relevant results, such as results from Francophone countries.

## Conclusions

This scoping review demonstrates that homeless people suffer from a range of physical and mental health problems and underutilise health services due to numerous accessibility and socio-economic barriers. Intervention models for complex and effective healthcare services and support for the homeless population are needed to help improve their health and wellbeing. Hospitals and clinics can be a key platform to address the health and welling of homeless people and to address homelessness as a whole, if various barriers can be minimised and utilisation improved. Thus, hospitals and clinics can be used as an opportunity to engage with homeless people, and help provide needs beyond regular hospital care, such as food, clothing, and other social support. This should be done through collaboration between hospitals/clinics and community-based homeless programs run by governments and NGOs. This could be effective through by ensuring stigma-free approaches that combine healthcare and social assistance as part of a package for homeless patients. The review also identified that studies did not focus on the interrelationship between homelessness and poor health and healthcare service utilisation in Africa, hence further research is needed. Future studies should also focus on utilisation of specific healthcare services that are important to the homeless adult population using mixed methods to take advantage of the strength of both methods achieve full understanding. Such services include accident and emergency, inpatient or hospitalisation, mental health, alcohol and drugs, and traditional or spiritual services. This would provide a stronger knowledge on the health problems and extent of use of different types of healthcare services.

### Funding

Six out of the 14 included studies reported receiving funding or financial support [11, 37, 38, 42, 44, 45]. Of these, two studies received financial support from the University of Gondar’s Research and Publications Office in Ethiopia [44, 45]. Two studies were funded by the Federal Ministry of Health, Ethiopia [11], and Ministry of Health of Mozambique [42]. Similarly, one included study was partially funded by the Amhara health regional bureau in Ethiopia [38]. One study conducted in South Africa was funded by both domestic and international organisations, including the National Department of Social Development, the Human Sciences Research Council, the Gauteng Department of Social Development, and the Swiss Agency for Development and Cooperation [37].

## Supplementary information

**Additional file 1: Table S1.** Sample Search Strategy-Medline. **Table S2.** Physical Health Problems. **Table S3.** Mental Health Problems. **Table S4.** Health service Utilisation.

## Data Availability

The materials used and/or analysed during the current review are available from the corresponding author on reasonable request.

## Abbreviation

MNS: Mental, Neurologic, and Substance Use
PTB: Pulmonary Tuberculosis
HIV: Human Immunodeficiency Virus
WHO: World Health Organisation
SRQ: Self-Reporting Questionnaire
PSQ: Psychosis Screening Questionnaire
MINI: Mini International Neuropsychiatric Interview
ASSIST: Alcohol, Smoking and Substance Involvement Screening Test
