# Supplementary material for: Health problems and healthcare service utilisation amongst homeless adults in Africa- a scoping review
Source: BMC Public Health. 2020 May 1;20:594. doi: 10.1186/s12889-020-08648-y (PMC7193394; doi:10.1186/s12889-020-08648-y)
Supplement: Supplementary file 1 — Additional file 1: Table S1. Sample Search Strategy-Medline. Table S2. Physical Health Problems. Table S3. Mental Health Problems. Table S4. Health service Utilisation. [file 12889_2020_8648_MOESM1_ESM.docx]

**Supplementary Table 1: Sample Search Strategy-Medline**

|  |  |
| --- | --- |
| 1 | exp homeless persons/ or homeless youth/ |
| 2 | ((Homeless* and (People or person* or youth or adult* or famil* or men or women)) or (Sleeping adj2 rough) or (street adj4 people) or roofless* or houseless*).ti. or ((Homeless* and (People or person* or youth or adult* or famil* or men or women)) or (Sleeping adj2 rough) or (street adj4 people) or roofless* or houseless*).ab. or ((Homeless* and (People or person* or youth or adult* or famil* or men or women)) or (Sleeping adj2 rough) or (street adj4 people) or roofless* or houseless*).kw. |
| 3 | (Unsheltered and (people or person* or youth or adult* or famil* or men or women)).ti. or (Unsheltered and (people or person* or youth or adult* or famil* or men or women)).ab. or (Unsheltered and (people or person* or youth or adult* or famil* or men or women)).kw. |
| 4 | (houseless* or roofless*).ti. or (houseless* or roofless*).ab. or (houseless* or roofless*).kw. |
| 5 | 1 or 2 or 3 or 4 |
| 6 | "diseases (non mesh)"/ or health/ or men's health/ or mental health/ or oral health/ or physical fitness/ or reproductive health/ or sexual health/ or suburban health/ or urban health/ or women's health/ |
| 7 | ORAL HEALTH/ or HEALTH STATUS/ or MATERNAL HEALTH/ or MENTAL HEALTH/ or MEN'S HEALTH/ |
| 8 | ((Health and (problem* or need* or condition* or status or physical or mental or poor)) or (Disease* or Infirmit* or comorbidit* or morbidit* or illness* or sickness* or ill health or mental illness*)).ti. or ((Health and (problem* or need* or condition* or status or physical or mental or poor)) or (Disease* or Infirmit* or comorbidit* or morbidit* or illness* or sickness* or ill health or mental illness*)).ab. or ((Health and (problem* or need* or condition* or status or physical or mental or poor)) or (Disease* or Infirmit* or comorbidit* or morbidit* or illness* or sickness* or ill health or mental illness*)).kw. |
| 9 | 6 or 7 or 8 |
| 10 | "health care (non mesh)"/ or health services/ or community health services/ or dental health services/ or dietary services/ or emergency medical services/ or health services for persons with disabilities/ or health services for the aged/ or mental health services/ or nursing care/ or nursing services/ or patient care/ or patient escort service/ or personal health services/ or pharmaceutical services/ or preventive health services/ or rehabilitation/ or reproductive health services/ or student health services/ or women's health services/ |
| 11 | (Health and (services or care or primary or facilit*) and (us* or utilisation or uptake or seek* or consum* or usage)).ti. or (Health and (services or care or primary or facilit*) and (us* or utilisation or uptake or seek* or consum* or usage)).ab. or (Health and (services or care or primary or facilit*) and (us* or utilisation or uptake or seek* or consum* or usage)).kw. |
| 12 | 10 or 11 |
| 13 | exp africa/ or exp africa, northern/ or exp "africa south of the sahara"/ |
| 14 | (Nigeria or Ghana or South Africa or Ethiopia or Kenya or Sudan or Egypt or Congo or Tanzania or Uganda or Zimbabwe or Zambia or Senegal or Sierra Leone or Gambia or Cameroon or Guinea or Libya or Namibia or Mali or Rwanda or Somalia or Mozambique or Liberia or Togo or Malawi or Burundi or Djibouti or Central African Republic or Chad or Lesotho).ti. or (Nigeria or Ghana or South Africa or Ethiopia or Kenya or Sudan or Egypt or Congo or Tanzania or Uganda or Zimbabwe or Zambia or Senegal or Sierra Leone or Gambia or Cameroon or Guinea or Libya or Namibia or Mali or Rwanda or Somalia or Mozambique or Liberia or Togo or Malawi or Burundi or Djibouti or Central African Republic or Chad or Lesotho).ab. or (Nigeria or Ghana or South Africa or Ethiopia or Kenya or Sudan or Egypt or Congo or Tanzania or Uganda or Zimbabwe or Zambia or Senegal or Sierra Leone or Gambia or Cameroon or Guinea or Libya or Namibia or Mali or Rwanda or Somalia or Mozambique or Liberia or Togo or Malawi or Burundi or Djibouti or Central African Republic or Chad or Lesotho).kw. |
| 15 | 15. 13 or 14 |
| 16 | 16. 5 and 9 and 15 |
| 17 | 5 and 12 and 15 |
| 18 | 16 or 17 |

**Supplementary Table 2: Physical Health Problems**

| **Reference** | **Country** | **Study Design** | **Sample Size** | **Sample type** | **Physical Health Problems** |
| --- | --- | --- | --- | --- | --- |
| 34. Abdu et al. (2013) | Nigeria | Quantitative/Descriptive | 65 | Homeless street people | - Visual impairment (24 subjects, including 23 due to cataracts and 1 due to glaucoma). - Hypertension (23 subjects), skin ulcer (2 subjects), scabies (1 subject). |
| 45. Moges et al. (2006) | Ethiopia | Quantitative-cross-sectional design | 404 | Street dwellers | - HIV Positive- 28 (6.9%) - Intestinal parasitic infection- 67.6% |
| 35. Moyo et al. (2015) | South Africa | Qualitative-exploratory design | 18 | Homeless persons with suspected mental illness | - Health problems among the homeless people include coughing and HIV |
| 36. Olufemi (1999) | South Africa | Qualitative design | 88 | Homeless street women | - High incidence of chronic and acute health problems (reported) - Common diseases based on intensity include tuberculosis; stomach diseases; diarrhoea and dysentery; bronchitis, headache; cold, cough, asthma, pneumonia, diabetes; arthritis; dental problems and eye infection. |
| 37. Seager & Tamasane (2010) | South Africa | Mixed | 1247 | Homeless street people and people in shelters | - Interview revealed common health problems such as TB, injuries, and skin diseases, HIV - Survey revealed that 19% of adults has a physical disability |
| 30. Wentzel & Voce (2012) | South Africa | Qualitative | 18 | Homeless persons in shelters | - Health problems reported include minor ailments, communicable and non-communicable diseases, physical injuries and trauma, and HIV |
| 38. Semunigus et al. (2016) | Ethiopia | Quantitative –community based cross-sectional study | 361 | Homeless individuals | - Smear positive Pulmonary TB- 2.6 % (n=9) - HIV- 22 (6.3 %) |
| 39. Khelil et al (2017) | Tunisia | Quantitative (descriptive, retrospective, and cross-sectional) | 152 | Cases of homeless mortality | - Cardiac diseases- 39 (44.6%) - Pulmonary diseases 33 (39.3%) |
| 43. Lohrmann et al. (2012) | South Africa | Quantitative (cross-sectional study) | 136 | Adults from homeless clinics | - HIV- 23.5% (n= 32) |

**Supplementary Table 3: Mental Health Problems**

| **Reference** | **Country** | **Study Design** | **Number of Participants** | **Summary Description of the Study**  **Population** | **Mental Health Problems** |
| --- | --- | --- | --- | --- | --- |
| 42. Gouveia et al. (2017) | Mozambique | Quantitative (descriptive) | 71 | Homeless people with apparent mental illness | - Schizophrenia- 46 (64.8%) - mental and behavioural disturbance- 21 (29.6%) - Intellectual disability- 4 (4.6%) |
| 37. Seager & Tamasane (2010) | South Africa | Mixed | 1247 | Homeless street people and people in shelters | - Depression- 58% - Mood disorder- 6% |
| 11. Ayano et al. (2017) | Ethiopia | Quantitative/community-based survey | 456 | Roofless people with overt and observable psychopathology | - mental neurologic and substance use (MNS) disorder- 92.11% - 85.29% of MNS patients had psychotic disorders |
| 40. De-Graft Aikins & Ofori-Atta (2007) | Ghana | Qualitative | 28 | Homeless adults living in squats | - Psychological stresses due to excessive thinking, worry and anxiety. |
| 41. Fekadu et al. (2014) | Ethiopia | Quantitative-cross-sectional design | 217 | Street homeless people 18 years and above | - Psychotic disorders- 41%. - Psychosis patients with schizophrenia- 88% - Bipolar disorder - 2.2% - Persistent wish to dies - 41.8% - Suicide attempt- 14.8% |

**Supplementary Table 4: Health service Utilisation**

| **Reference** | **Country** | **Study Design** | **Number of Participants** | **Summary Description of the Study**  **Population** | **Results on health service utilization (type and frequency)** |
| --- | --- | --- | --- | --- | --- |
| 36. Olufemi (1999) | South Africa | Qualitative design | 88 | Homeless street women | - Underutilisation of hospital and clinic services |
| 37. Seager & Tamasane (2010) | South Africa | Mixed | 1247 | Homeless street people and people in shelters | - Utilisation of hospital services- 25.3% - Utilisation of complementary services- 5.8% - Traditional healing services -2.9% |
| 30. Wentzel & Voce (2012) | South Africa | Qualitative | 18 | Homeless persons in shelters | - Underutilisation of hospital services |
| 44. Megabiaw (2012) | Ethiopia | Quantitative (cross-sectional) | 204 | Street women | - Lifetime contraceptive usage - 96 (47.1%) - Current contraceptive usage- 70 (34.3%). |
| 35. Abdul et al. (2013) | Nigeria | Quantitative | 65 | Homeless street people | - Utilisation of hospital services – 15% - Traditional healing services- 17% |
